# Preoperative Melatonin for Women Undergoing Cesarean Section: A Systematic Review and Updated Meta-Analysis of Randomized Controlled Trials with Trial Sequential Analysis

**DOI:** 10.3390/diseases14050181

**Published:** 2026-05-20

**Authors:** Zlatko Kirovakov, Andriana Jovanovska-Kirovakova, Angel Yordanov, Eva Tsoneva, Monika Obreykova, Plamen Penchev

**Affiliations:** 1 Faculty for Public Health and Health Care, Burgas State University “Prof. d-rAsen Zlatarov”, 8000 Burgas, Bulgaria; zlatko-kirovakov@uniburgas.bg (Z.K.); dr_jovanovska@abv.bg (A.J.-K.); monika_obrejkova@abv.bg (M.O.); 2 Department of Gynecological Oncology, Medical Universiy Pleven, 5800 Pleven, Bulgaria; 3 Department of Gynecology, Military Medical Academy–Sofia, 1000 Sofia, Bulgaria; dretsoneva@gmail.com; 4 Faculty of Medicine, Medical University of Plovdiv, 4000 Plovdiv, Bulgaria; sonaonetrick@abv.bg

**Keywords:** melatonin, cesarean section, meta-analysis, pregnant women, postoperative pain

## Abstract

**Introduction:** Effective perioperative management in cesarean section remains essential to optimize maternal outcomes. Melatonin (M) has been proposed as a potential adjunct due to its analgesic, anxiolytic, and antiemetic properties; however, evidence from randomized controlled trials (RCTs) remains inconsistent. This meta-analysis aimed to evaluate the efficacy and safety of preoperative melatonin compared with placebo in women undergoing cesarean section. **Methods:** A systematic search was conducted in PubMed, Scopus, and Cochrane from inception to 15 March 2026 for studies evaluating pregnant women undergoing elective cesarean section receiving preoperative melatonin versus placebo (P) (PROSPERO “CRD420261355468”). Heterogeneity was assessed using the I^2^ statistic and Cochrane Q test. Risk ratios (RRs) and standardized mean differences (SMDs) were computed using a restricted maximum-likelihood estimator random-effects method. Trial Sequential Analysis (TSA) was performed to assess the robustness and sufficiency of the evidence. **Results:** Seven RCTs were included with 552 patients (melatonin: 278; placebo: 274). Preoperative melatonin significantly reduced opioid consumption in the overall pooled analysis (RR 0.31, 95% CI 0.12 to 0.80; *p* = 0.030; I^2^ = 50%), and TSA supported the robustness of this opioid-sparing finding under the selected assumptions. Postoperative pain scores were also significantly lower in the melatonin group (SMD −2.10, 95% CI −2.43 to −1.78; *p* < 0.01; I^2^ = 22%). The incidence of postoperative nausea showed a trend toward reduction in the conventional meta-analysis (RR 0.49, 95% CI 0.23–1.04; *p* = 0.057; I^2^ = 34%); although TSA suggested a possible benefit, this finding should be considered exploratory. No significant difference was observed in intraoperative blood loss (SMD −0.33, 95% CI −1.53 to 0.88; *p* = 0.60; I^2^ = 94%). **Conclusions:** Preoperative melatonin may be a promising adjunct in cesarean section, particularly for reducing postoperative pain and overall opioid consumption. TSA findings support the opioid-sparing result under selected assumptions, while the possible effect on postoperative nausea remains exploratory. Further high-quality trials are warranted before routine clinical implementation can be recommended.

## 1. Introduction

Cesarean section is one of the most commonly performed surgical procedures worldwide, with a continuously rising incidence. Effective perioperative management remains essential to ensure optimal maternal recovery, particularly in terms of postoperative pain control, minimization of opioid use, and reduction in anesthesia-related adverse effects such as nausea. Inadequate pain control following cesarean delivery is associated with delayed mobilization, impaired maternal–neonatal bonding, and an increased risk of chronic pain development [1,2]. Although multimodal analgesia strategies are widely implemented, the search for safe and effective adjuncts that can enhance analgesia while limiting opioid-related side effects remains ongoing [3].

Melatonin is an endogenous indoleamine primarily produced by the pineal gland, known for its role in regulating circadian rhythms. Beyond its chronobiological function, melatonin has demonstrated analgesic, anxiolytic, anti-inflammatory, and opioid-sparing properties in human perioperative studies. These properties are clinically relevant in cesarean delivery, where postoperative pain control must be balanced against maternal sedation, nausea, opioid exposure, breastfeeding, early mobilization, and maternal–neonatal interaction. However, direct evidence in parturients remains limited, and findings from non-obstetric or experimental studies should be extrapolated cautiously. These effects are thought to be mediated through modulation of central pain pathways, interaction with opioid receptors, and reduction in oxidative stress and inflammatory mediators [4,5]. Given its favorable safety profile and low cost, melatonin has emerged as a promising perioperative adjunct across various surgical settings, including obstetric anesthesia [6].

Several randomized controlled trials (RCTs) have evaluated the role of preoperative melatonin in women undergoing cesarean section, with some reporting reduced postoperative pain scores, decreased opioid consumption, and improved perioperative comfort [7,8]. However, the findings have been inconsistent, particularly regarding secondary outcomes such as postoperative nausea and intraoperative blood loss [6,9,10]. A recent meta-analysis attempted to synthesize the available evidence regarding preoperative melatonin in women undergoing cesarean section; however, several methodological issues limit the certainty of its conclusions [11]. In particular, that analysis included one conference abstract with insufficient extractable data and another study that could not be reliably verified because of an untraceable DOI. The inclusion of non-verifiable or incompletely reported studies may compromise reproducibility, increase the risk of data extraction errors, and reduce confidence in pooled estimates. Therefore, an updated synthesis applying stricter eligibility criteria, transparent data verification, and additional robustness analyses is warranted.

Importantly, since the publication of the previous meta-analysis, new randomized evidence has become available, and methodological standards for evidence synthesis have evolved, emphasizing transparency, reproducibility, and rigorous data verification. Furthermore, conventional meta-analytic approaches may be prone to random errors when cumulative sample sizes are small, highlighting the importance of advanced techniques such as Trial Sequential Analysis (TSA) to assess the sufficiency and reliability of the evidence [12,13].

Therefore, the present study aimed to provide an updated and methodologically rigorous systematic review and meta-analysis of RCTs evaluating preoperative melatonin in women undergoing cesarean section. By incorporating newly published studies, reassessing previously included trials, and applying TSA, this study seeks to clarify the efficacy and safety profile of melatonin and determine whether the current evidence is sufficient to support its routine clinical use.

## 2. Methods

### 2.1. Eligibility Criteria

This systematic review and meta-analysis followed the Cochrane Handbook for Systematic Reviews of Interventions and the Preferred Reporting Items for Systematic Reviews and Meta-Analysis Statement [14,15]. This meta-analysis did not require Institutional Review Board approval because it used data from previously published and publicly available articles. Studies that met all the following criteria were included in the meta-analysis: (1) pregnant women undergoing elective cesarean section under any type of anesthesia (regional or general); (2) preoperative administration of melatonin before skin incision or before induction/administration of anesthesia, with clearly reported dose, timing, and route of administration (Because available RCTs used different regimens, no restriction was applied according to melatonin dose or timing; however, these variables were extracted and explored descriptively and, when possible, through subgroup analyses. The reported routes of administration included oral and sublingual melatonin); (3) studies with the control group receiving placebo or standard care without melatonin; (4) studies reporting at least one of the outcomes from postoperative pain scores, analgesic (opioid) consumption, intraoperative blood loss, or postoperative nausea; (5) outcomes assessed during the perioperative or postoperative period, defined as intraoperative time up to hospital discharge or a predefined follow-up period; and (6) randomized placebo-controlled trials (RCTs). Trials were eligible regardless of whether cesarean section was performed under regional or general anesthesia, provided that the anesthesia protocol was clearly described and balanced between treatment groups. Anesthesia type was not used as a formal inclusion criterion because the available evidence base was small and overly restrictive criteria would have excluded otherwise relevant randomized trials. Nevertheless, anesthesia protocols were extracted where available and considered as a potential source of clinical heterogeneity, because anesthetic agents and neuraxial opioid use may influence postoperative pain, nausea, and opioid consumption. Studies were excluded if they met one of the following criteria: (1) studies not involving cesarean section or including mixed surgical populations where cesarean-specific data could not be extracted separately; (2) studies evaluating melatonin administered exclusively intraoperatively or postoperatively, or studies in which the timing of melatonin administration relative to anesthesia or surgical incision was not clearly reported (“Clearly defined preoperative administration” was defined as melatonin administered before the start of anesthesia or before surgical incision, with sufficient information to determine that administration occurred before the operative intervention.); (3) trials lacking a placebo or appropriate control group; (4) overlapping populations; (5) case reports or series, editorials, letters, conference abstracts without full-text availability, and animal or in vitro models; and (6) studies with insufficient or non-extractable data for the outcomes of interest. This systematic review and meta-analysis was registered with the International Prospective Register of Systematic Reviews (PROSPERO) under the ID “CRD420261355468”.

### 2.2. Search Strategy and Data Extraction

We systematically searched PubMed, Scopus, and Cochrane Central from inception to 15 March 2026 with the following search strategy: (Melatonin OR “pineal hormone” OR NSC-113928) AND (“Cesarean section” OR “Caesarean section” OR “abdominal delivery” OR “abdominal deliveries” OR “C-section” OR “C section” OR “postcesarean section”) AND (randomized controlled trial[pt] OR controlled clinical trial[pt] OR clinical trials as topic[mesh:noexp] OR trial[ti] OR random*[tiab] OR placebo*[tiab]). Only studies published in the English language were considered eligible, while gray literature sources were excluded from the analysis. In addition, the reference lists of all included articles were manually screened to identify any further relevant studies. Data extraction was independently conducted by two reviewers (Z.K. and P.P.) using predefined eligibility criteria, standardized quality assessment procedures, and the platform Rayyan [16]. Any discrepancies arising during the process were resolved through discussion and mutual agreement.

### 2.3. Endpoints and Subgroup Analyses

The meta-analysis included postoperative pain, nausea and opioid usage, and intraoperative blood loss endpoints. Additionally, we conducted a subgroup analysis based on the melatonin dosage and risk of bias. For postoperative pain intensity, the primary time point was defined as pain score at 24 h after cesarean section. When 24 h data were not available, the closest reported time point within the 12–24 h postoperative interval was extracted. If multiple postoperative pain measurements were reported within a study, only the predefined time point was used in the primary meta-analysis to avoid double-counting participants. When sufficient data were available, separate time-point analyses were planned for early postoperative pain, intermediate pain, and 24 h pain.

### 2.4. Quality Assessment

The risk of bias was evaluated using the Cochrane Collaboration Risk of Bias 2 (RoB 2) tool for randomized intervention studies, which classifies studies as having low risk, some concerns, or high risk of bias [17]. These assessments were performed independently by two reviewers (Z.K. and P.P.), with disagreements settled by consensus. Publication bias was explored through contour-enhanced funnel plots combined with the trim-and-fill approach, facilitating improved interpretation of asymmetry in relation to statistical significance thresholds. Additional approaches, including *p*-curve and *p*-uniform analyses, could not be applied because exact *p*-values or test statistics were not consistently reported across all included studies. In accordance with Cochrane recommendations, Egger’s regression test was not performed because the meta-analysis included fewer than 10 studies [15].

### 2.5. Statistical Analysis

For continuous outcomes, effect sizes were calculated as standardized mean differences (SMDs) with 95% confidence intervals (CIs) using the inverse-variance method and a random-effects model estimated by restricted maximum likelihood [18,19]. For dichotomous outcomes, risk ratios (RRs) with 95% CIs were derived using the Mantel–Haenszel method [20]. A random-effects framework was applied uniformly across all outcomes to account for both methodological and demographic heterogeneity among the included studies. Statistical heterogeneity was assessed using Cochran’s Q test and the I^2^ statistic. A two-sided *p*-value below 0.05 was considered statistically significant. Subgroup analyses stratified by melatonin dose and risk-of-bias category were conducted to explore potential sources of heterogeneity and to assess the robustness of the findings across clinically and methodologically relevant strata. In addition, leave-one-out (LOO) sensitivity analyses were performed to evaluate the stability of the pooled estimates. Because possible overlap could not be fully excluded for studies from the same author groups with identical or highly similar sample sizes and baseline characteristics, we also performed post hoc overlap-sensitivity analyses. Specifically, we repeated the primary pooled analyses after excluding one study from each potentially overlapping pair—Alkhfaji 2023/Alkhfaji 2024 and Khezri 2016/Khezri 2019. As a conservative worst-case approach, we additionally repeated the analyses excluding both potentially duplicate later reports simultaneously. These analyses were considered exploratory and were used to assess whether the direction and statistical interpretation of the main findings were dependent on potentially overlapping study populations. A Baujat plot was also constructed to identify studies with the greatest contribution to between-study heterogeneity and overall effect size influence. This graphical method illustrates each study’s contribution to heterogeneity on the x-axis and its influence on the pooled result on the y-axis, thereby facilitating the detection of outliers or highly influential studies. All analyses were carried out using R version 4.3.1 with the metafor and meta packages [21].

### 2.6. Trial Sequential Analysis (TSA)

To further examine the robustness of the findings and minimize the risks of type I and type II errors associated with sparse data and repeated significance testing, Trial Sequential Analysis (TSA) was performed. The analysis was conducted using Trial Sequential Analysis Viewer version 0.9.5.10 Beta, applying a two-sided alpha level of 5% and statistical power of 80% [22]. The required information size (RIS) was estimated based on an anticipated relative risk reduction of 18%, a control event rate of 20%, and heterogeneity adjustment under a random-effects model. Adjusted cumulative Z-curves were then generated to determine whether the cumulative evidence crossed the TSA monitoring boundaries for significance or achieved the RIS threshold. The anticipated relative risk reduction and control event rate were selected based on clinically plausible assumptions and event rates observed in the included trials. Because TSA can be sensitive to these assumptions, especially when the number of included studies is small, TSA findings were interpreted as supportive rather than definitive. For outcomes in which conventional meta-analysis and TSA provided discordant conclusions, the TSA result was considered exploratory.

## 3. Results

### 3.1. Study Selection and Baseline Characteristics

**Figure 1:**
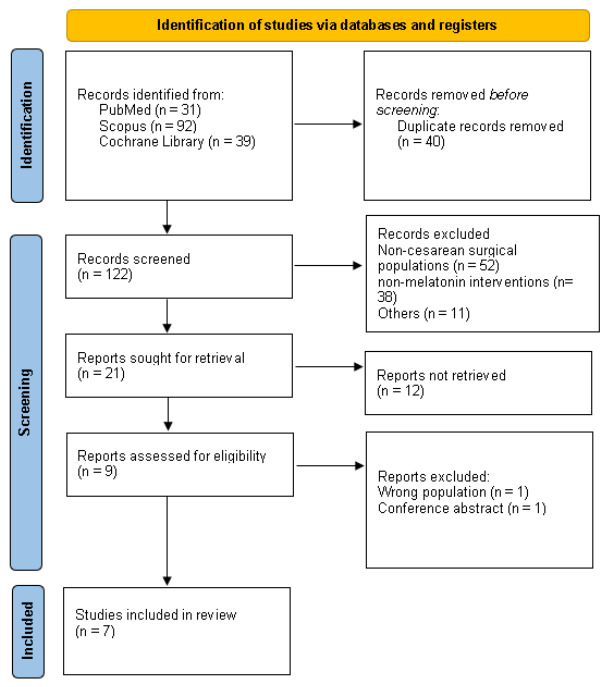
PRISMA flow chart and study selection process.

The search strategy yielded a total of 162 results. After removing duplicate records during title and abstract screening, 101 records were excluded because they were unrelated to the research question. The most common reasons were non-cesarean surgical populations, non-melatonin interventions, non-randomized designs, animal or experimental studies, reviews, and studies not reporting relevant perioperative outcomes. The remaining nine studies were fully reviewed to determine whether they met the inclusion and exclusion criteria (Figure 1). Of the studies included in the previous meta-analysis, two were excluded: one conference abstract due to insufficient data for extraction and one study that could not be verified due to an untraceable DOI. Seven studies were included, with 552 patients [7,8,23,24,25,26,27]. Of those, 278 patients (50%) were treated preoperatively with Melatonin. The mean age of the population was 28 ± SD years. Study characteristics are presented in Table 1 and Table 2.

Baseline characteristics revealed highly similar or identical values for some studies conducted by the same author groups, particularly Alkhfaji 2023/Alkhfaji 2024 and Khezri 2016/Khezri 2019. Although the available publications did not explicitly state that the cohorts overlapped, the similarity in sample size and baseline characteristics raised the possibility of partial or complete overlap. Therefore, these pairs were considered potentially overlapping in sensitivity analyses. The primary analysis retained all eligible published RCTs, whereas overlap-sensitivity analyses were performed to evaluate whether the pooled estimates were materially affected by excluding one study from each potentially overlapping pair.

### 3.2. Pooled Analyses of the Included Studies

#### 3.2.1. Postoperative Pain Intensity

**Figure 2:**
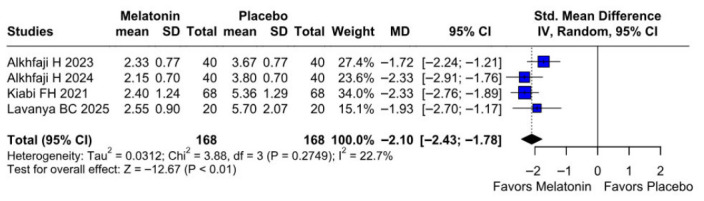
Forest plot comparing the effect of preoperative melatonin versus placebo on postoperative pain levels. The pooled SMD indicates a significant reduction in pain levels favoring the melatonin treatment [8,23,24,27].

**Figure 3:**
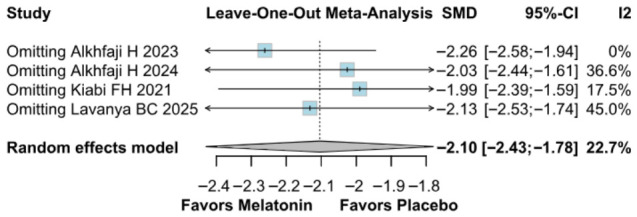
In terms of pain levels, the overall effect size remained consistent across all iterations and the result remained significant in all cases. Heterogeneity remained low throughout all iterations [8,23,24,27].

**Figure 4:**
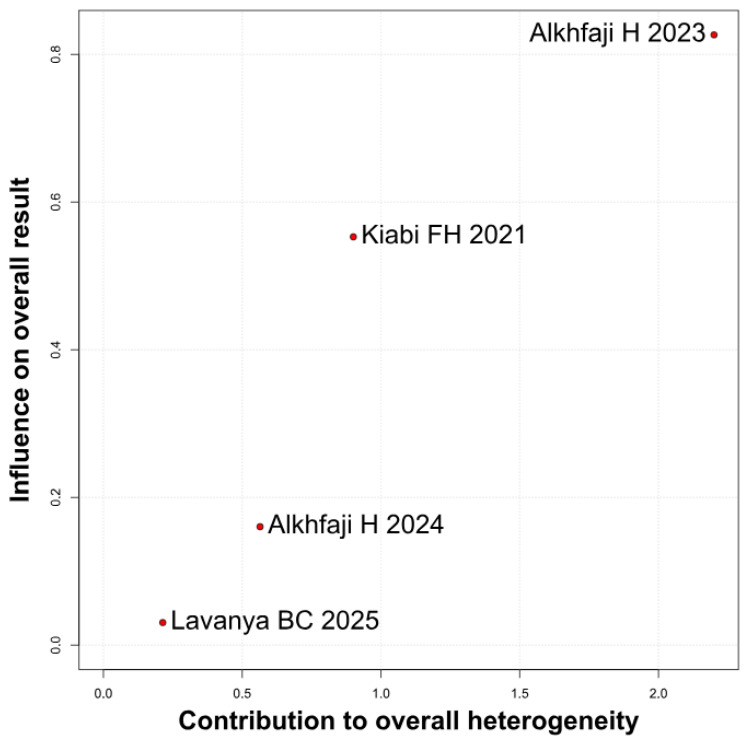
Baujat plot. The study by Alkhfaji H 2023 was discovered as potentially influential, contributing substantially to the overall result and heterogeneity [8,23,24,27].

The primary pain analysis used the predefined postoperative time point described in the Methods section. When studies reported multiple postoperative pain scores, only one time point per study was extracted for the primary analysis to avoid double-counting participants. Therefore, the pooled SMD reflects differences in pain intensity at the selected postoperative time horizon rather than an average across all reported time points. Preoperative melatonin significantly reduced postoperative pain intensity (SMD −2.10, 95% CI −2.43 to −1.78; *p* < 0.01; I^2^ = 22%) (Figure 2). A leave-one-out sensitivity analysis was conducted to assess the robustness of the pooled findings. The overall effect estimate remained stable throughout all iterations and retained statistical significance in every case (SMD −2.10, 95% CI −2.43 to −1.78; *p* < 0.01; I^2^ = 22%) (Figure 3). These findings indicate that no individual study exerted a disproportionate influence on the overall result. The Baujat plot suggested that the study by Alkhfaji H. (2023) was potentially influential, contributing substantially to both the pooled effect estimate and observed heterogeneity (Figure 4).

#### 3.2.2. Opioid Consumption

**Figure 5:**
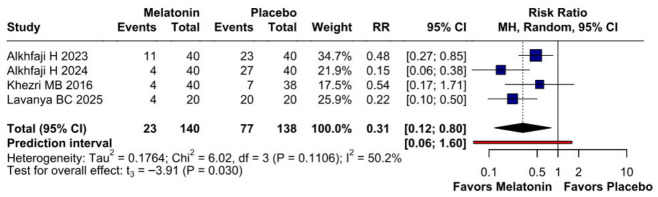
Forest plot comparing the effect of preoperative melatonin versus placebo on postoperative opioid consumption. The pooled RR indicates a significant reduction in opioid consumption favoring the melatonin treatment [7,23,24,27].

**Figure 6:**
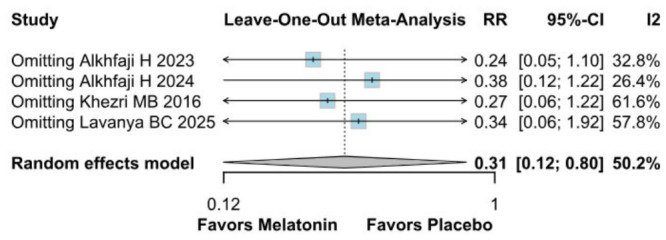
In terms of opioid consumption, the overall effect size remained consistent across all iterations, and the result remained significant in all cases. Heterogeneity remained moderate throughout all iterations [7,23,24,27].

**Figure 7:**
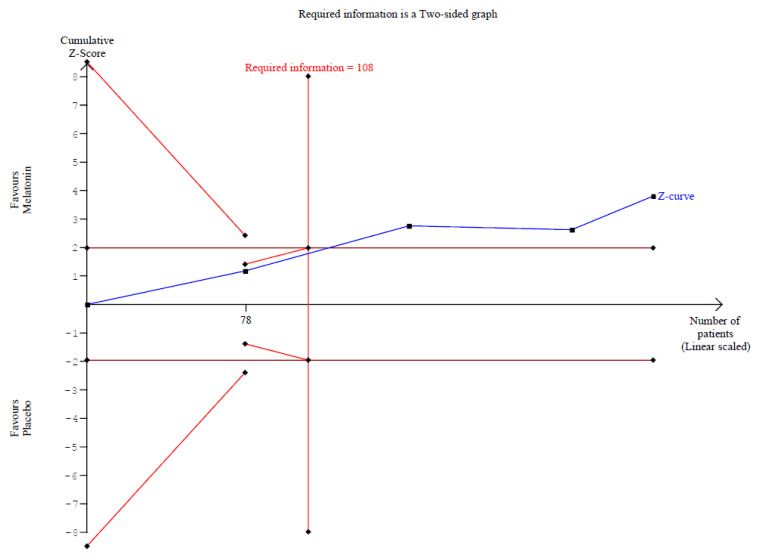
TSA for opioid consumption comparing melatonin versus placebo. TSA suggested that the cumulative evidence may be compatible with benefit under the assumed parameters [7,23,24,27].

**Figure 8:**
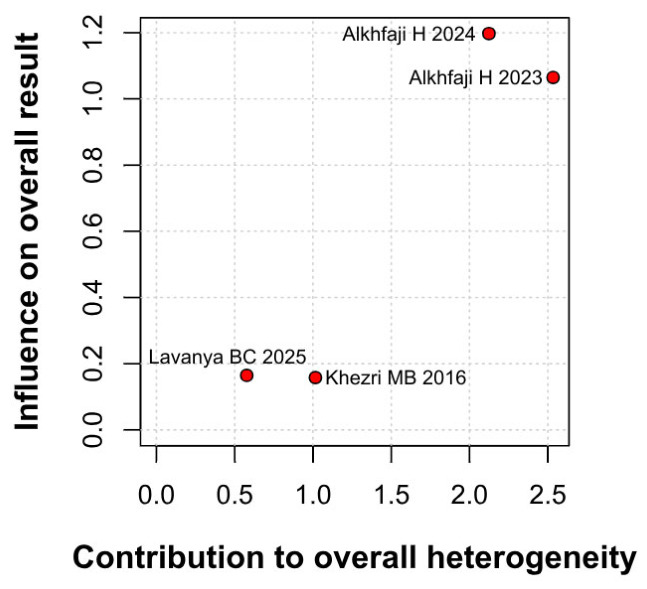
Baujat plot. The studies by Alkhfaji H 2023 and Alkhfaji H 2024 were identified as potentially influential, contributing substantially to the overall result and heterogeneity [7,23,24,27].

A significant reduction in opioid consumption was found after preoperative melatonin administration (RR 0.31, 95% CI 0.12 to 0.80; *p* = 0.030; I^2^ = 50%) (Figure 5). A leave-one-out sensitivity analysis was similarly performed to evaluate the robustness of this outcome. The pooled effect size remained stable across all iterations and continued to demonstrate statistical significance (RR 0.31, 95% CI 0.12 to 0.80; *p* = 0.030; I^2^ = 50%) (Figure 6), suggesting the absence of undue influence from any single study. Furthermore, TSA suggested that the cumulative evidence may be compatible with benefit under the assumed parameters; however, because TSA results are sensitive to assumptions regarding anticipated effect size and control event rate, these findings should be interpreted cautiously (Figure 7). According to the Baujat plot, the studies by Alkhfaji H. (2023) and Alkhfaji H. (2024) appeared to be the most influential contributors to both the pooled effect and heterogeneity (Figure 8).

#### 3.2.3. Postoperative Nausea

**Figure 9:**
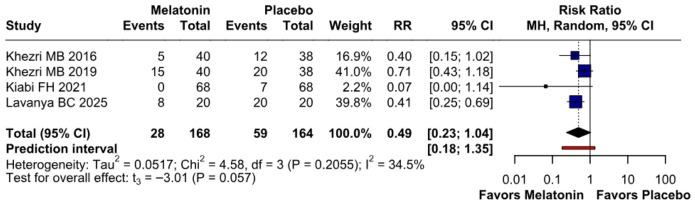
Melatonin administration showed a trend towards reducing postoperative nausea compared to placebo; however, the *p*-value did not reach statistical significance [7,8,26,27].

**Figure 10:**
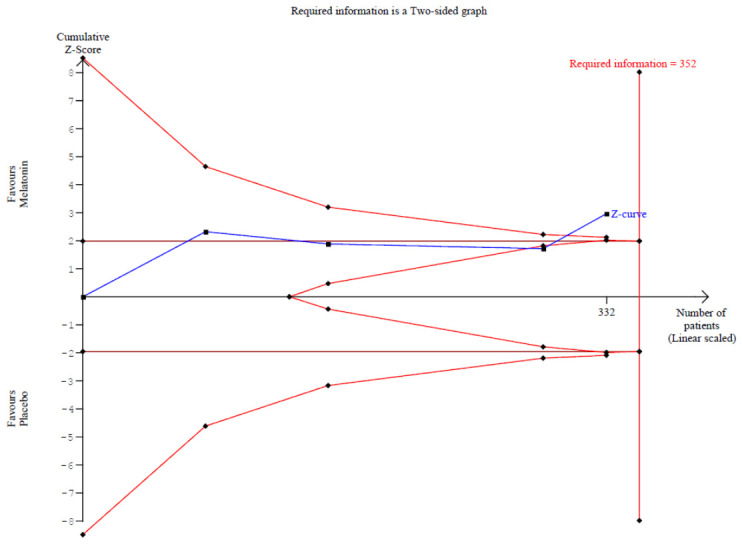
TSA for postoperative nausea reduction comparing melatonin and placebo. The cumulative Z-curve (blue line) crosses the trial sequential monitoring boundary and almost meets the required information size (RIS = 352) [7,8,26,27].

**Figure 11:**
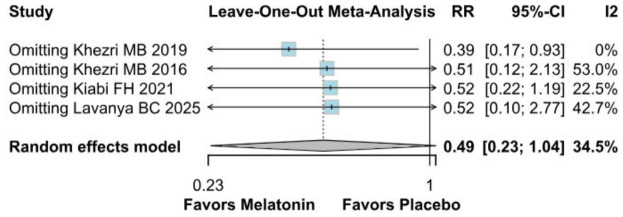
In terms of postoperative nausea, the overall effect size remained consistent across all iterations, and the result remained non-significant in all cases. Heterogeneity remained moderate throughout all iterations [7,8,26,27].

**Figure 12:**
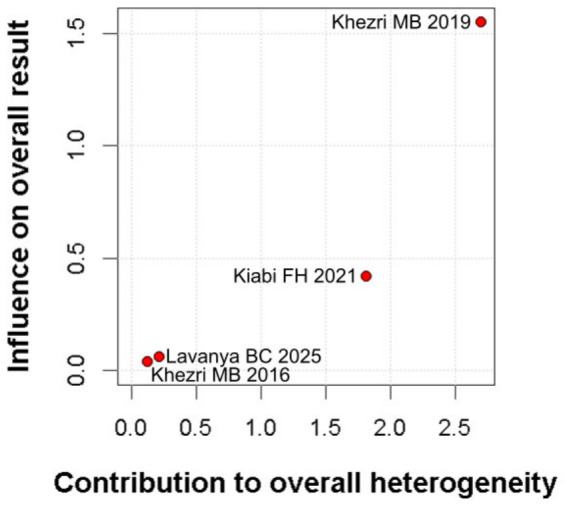
Baujat plot. The study by Khezri MB 2019 was discovered as potentially influential, contributing substantially to the overall result and heterogeneity [7,8,26,27].

The incidence of postoperative nausea showed a trend toward reduction (RR 0.49, 95% CI 0.23–1.04; *p* = 0.057; I^2^ = 34%) (Figure 9). TSA crossed the monitoring boundary under the selected assumptions; however, because the conventional meta-analysis narrowly missed statistical significance and TSA is sensitive to assumptions regarding anticipated effect size and control event rate, this finding should be interpreted cautiously and considered exploratory (Figure 10). A leave-one-out sensitivity analysis was carried out to test the stability of the results. Across all iterations, the pooled effect estimate remained consistent (RR 0.49, 95% CI 0.23–1.04; *p* = 0.057; I^2^ = 34%) (Figure 11), indicating that no single study disproportionately affected the overall findings. The Baujat plot identified the study by Khezri M.B. (2019) as a potentially influential source of both heterogeneity and effect size contribution (Figure 12).

#### 3.2.4. Intraoperative Blood Loss

**Figure 13:**
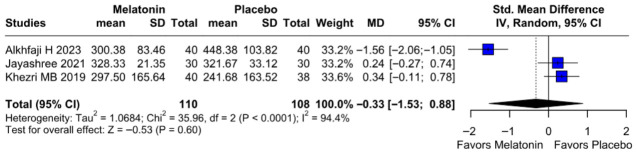
Forest plot comparing the effect of preoperative melatonin versus placebo on intraoperative blood loss. The pooled SMD indicates a non-significant reduction in blood loss [23,25,26].

**Figure 14:**
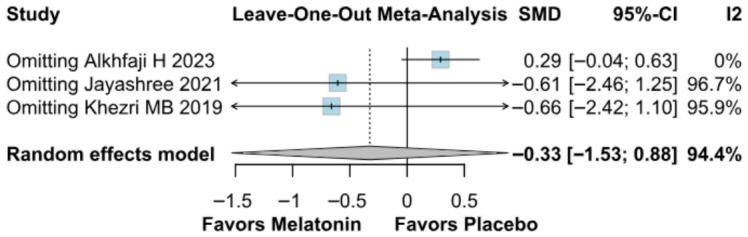
In terms of intraoperative blood loss, the overall effect size remained consistent across all iterations, and the result remained non-significant in all cases. Heterogeneity remained high throughout all iterations [23,25,26].

**Figure 15:**
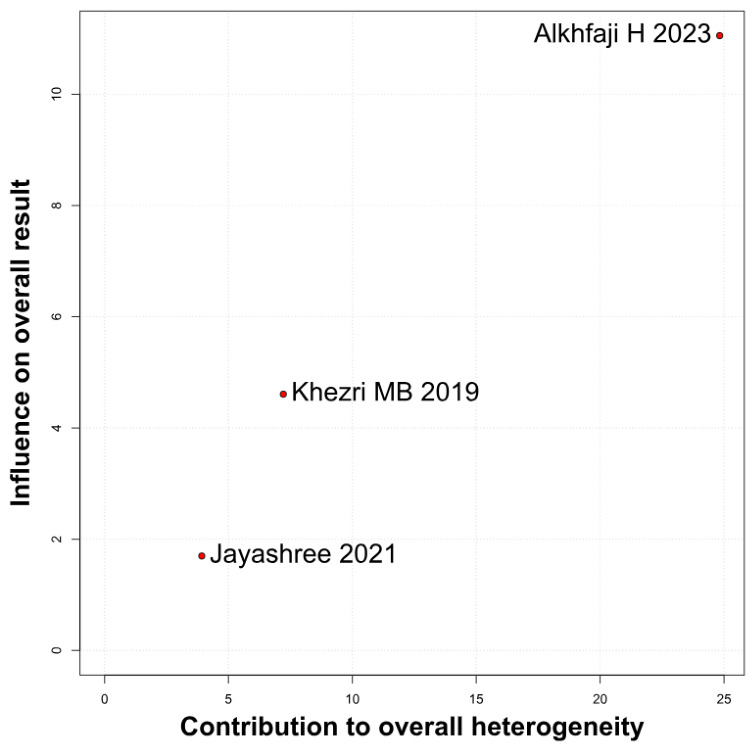
Baujat plot. The study by Alkhfaji H 2023 was discovered as potentially influential, contributing substantially to the overall result and heterogeneity [23,25,26].

No significant difference was observed in intraoperative blood loss between melatonin and placebo (SMD −0.33, 95% CI −1.53 to 0.88; *p* = 0.60; I^2^ = 94%) (Figure 13). To assess the robustness of this outcome, a leave-one-out sensitivity analysis was performed. The pooled effect estimate remained largely unchanged across all iterations (SMD −0.33, 95% CI −1.53 to 0.88; *p* = 0.60; I^2^ = 94%) (Figure 14), suggesting that no individual study had a disproportionate impact on the overall result. However, the Baujat plot indicated that the study by Alkhfaji H. (2023) was a major contributor to both the overall effect estimate and the substantial heterogeneity observed (Figure 15).

### 3.3. Sensitivity Analyses for Potentially Overlapping Study Populations

#### 3.3.1. Postoperative Pain Levels

**Figure 16:**
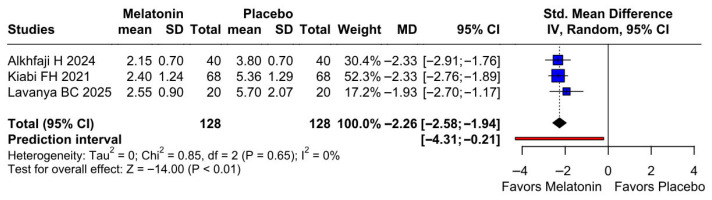
Sensitivity analysis for postoperative pain excluding Alkhfaji 2023. After exclusion of this potentially influential study, preoperative melatonin remained associated with significantly lower postoperative pain scores compared with placebo (SMD −2.26, 95% CI −2.58 to −1.94; *p* < 0.01), with no observed heterogeneity (I^2^ = 0%) [8,24,27].

Because Alkhfaji 2023 was identified as potentially influential and possible overlap with Alkhfaji 2024 could not be fully excluded, we performed an additional sensitivity analysis excluding Alkhfaji 2023. The pooled effect remained statistically significant and continued to favor melatonin, with an SMD of −2.26 (95% CI −2.58 to −1.94; *p* < 0.01). Heterogeneity was eliminated in this analysis (I^2^ = 0%), suggesting that the significant reduction in postoperative pain was not dependent on the inclusion of Alkhfaji 2023 (Figure 16).

#### 3.3.2. Postoperative Nausea

**Figure 17:**
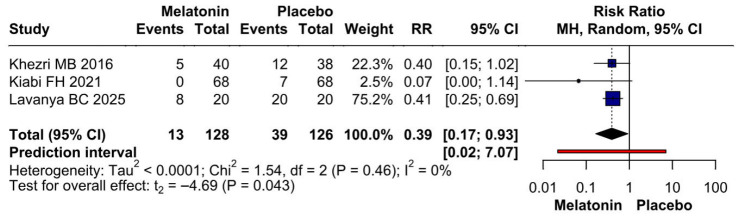
Sensitivity analysis for postoperative nausea excluding Khezri MB 2019. After exclusion of this potentially influential study, preoperative melatonin was associated with a statistically significant reduction in postoperative nausea compared with placebo (RR 0.39, 95% CI 0.17 to 0.93; *p* = 0.043), with no observed heterogeneity (I^2^ = 0%). However, the wide prediction interval crossing the null value indicates that the result should be interpreted cautiously [8,26,27].

Because Khezri MB 2019 was identified as potentially influential and possible overlap with Khezri MB 2016 could not be fully excluded, we performed an additional sensitivity analysis excluding Khezri MB 2019. After exclusion of this study, preoperative melatonin was associated with a statistically significant reduction in postoperative nausea (RR 0.39, 95% CI 0.17 to 0.93; *p* = 0.043), and heterogeneity was reduced to 0%. However, the prediction interval remained wide and crossed the null value (0.02 to 7.07), indicating substantial uncertainty regarding the expected effect in future studies. Therefore, this sensitivity finding should be interpreted cautiously and considered exploratory (Figure 17).

#### 3.3.3. Opioid Consumption

**Figure 18:**
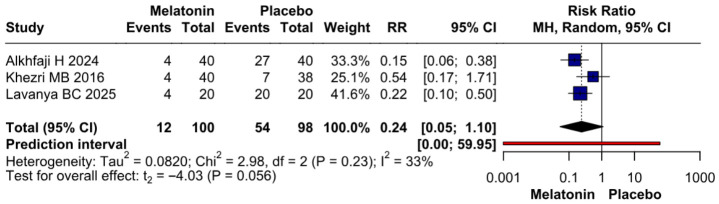
Sensitivity analysis for postoperative opioid consumption excluding Alkhfaji H 2023. After exclusion of this potentially influential study, the direction of effect remained in favor of melatonin, but the pooled result no longer reached conventional statistical significance (RR 0.24, 95% CI 0.05 to 1.10; *p* = 0.056; I^2^ = 33%). The wide prediction interval crossing the null value indicates substantial uncertainty [7,24,27].

Because Alkhfaji H 2023 was identified as potentially influential and possible overlap with Alkhfaji H 2024 could not be fully excluded, we performed an additional sensitivity analysis excluding Alkhfaji H 2023. After exclusion of this study, the direction of effect for postoperative opioid consumption remained in favor of melatonin, but the pooled estimate no longer reached conventional statistical significance (RR 0.24, 95% CI 0.05 to 1.10; *p* = 0.056; I^2^ = 33%). The prediction interval was wide and crossed the null value, indicating considerable uncertainty. Therefore, the opioid-sparing finding should be interpreted cautiously and may be sensitive to the inclusion of potentially influential studies (Figure 18).

### 3.4. Subgroup Analyses

#### 3.4.1. Melatonin Dosage

##### Postoperative Pain Levels

**Figure 19:**
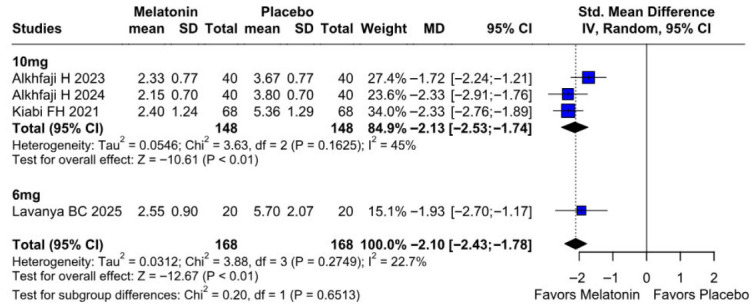
Forest plot of pain scores by melatonin dosage. Subgroup analysis shows significant pain reduction in both 6 mg (SMD −1.93; *p* < 0.01) and 10 mg (SMD −2.93; *p* < 0.01) groups. The test for subgroup differences was non-significant (*p* = 0.6513). The test for subgroup differences was non-significant (*p* = 0.6513), indicating no evidence of a statistically significant difference between the 6 mg and 10 mg subgroups [8,23,24,27].

In the dose-based subgroup analysis, both 6 mg and 10 mg melatonin significantly reduced postoperative pain compared with placebo. The magnitude of effect appeared numerically larger in the 10 mg subgroup than in the 6 mg subgroup; however, the test for subgroup differences was not statistically significant (*p* = 0.6513). Therefore, the available evidence does not demonstrate a clear dose–response relationship for postoperative pain reduction between 6 mg and 10 mg. For postoperative nausea, both dose subgroups showed a trend toward benefit, but neither reached conventional statistical significance. For intraoperative blood loss, subgroup differences were statistically significant; however, this finding should be interpreted cautiously because of the small number of studies, high heterogeneity, and potential differences in study protocols (Figure 19).

##### Opioid Consumption

**Figure 20:**
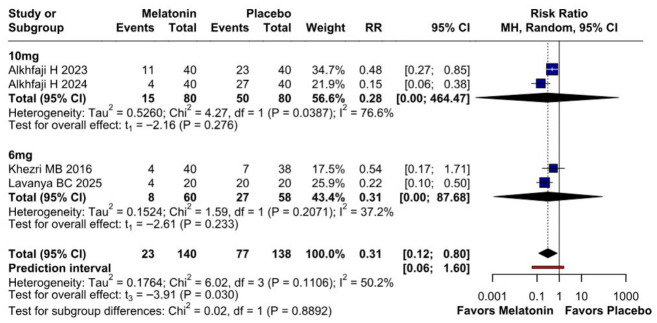
Subgroup analysis of postoperative opioid consumption by melatonin dose. Neither the 6 mg subgroup (RR 0.31; *p* = 0.233) nor the 10 mg subgroup (RR 0.28; *p* = 0.276) reached statistical significance individually. The test for subgroup differences was not statistically significant (*p* = 0.8892), indicating no evidence of a statistically significant difference between dose subgroups. Therefore, dose-specific opioid findings should be interpreted cautiously [23,24,26,27].

The subgroup analysis for postoperative opioid consumption showed a non-significant reduction for both the 10 mg subgroup (RR 0.28; *p* = 0.276) and the 6 mg subgroup (RR 0.31; *p* = 0.233). The test for subgroup differences was not statistically significant (*p* = 0.8892), indicating no evidence of a statistically significant difference between the two dose subgroups. However, because neither individual dose subgroup reached statistical significance, the opioid-sparing effect should be interpreted primarily at the overall pooled level rather than as reliable evidence of benefit within each dose category (Figure 20).

##### Postoperative Nausea

**Figure 21:**
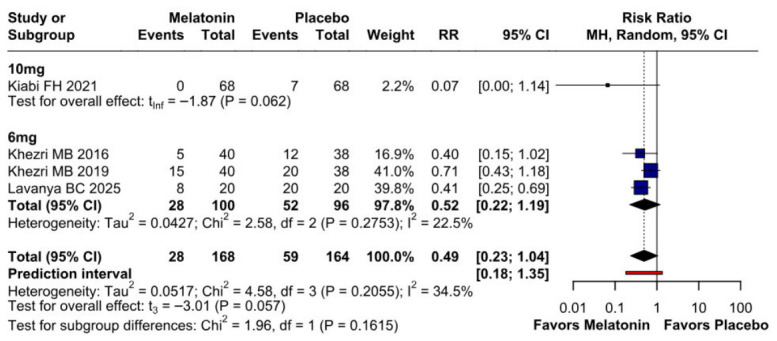
Subgroup analysis of postoperative nausea by melatonin dose. Neither the 10 mg subgroup (RR 0.07; 95% CI 0.00 to 1.14; *p* = 0.062) nor the 6 mg subgroup (RR 0.52; 95% CI 0.22 to 1.19; *p* = 0.057) reached statistical significance. Although both point estimates favored melatonin, the confidence intervals crossed the null value, indicating that dose-specific findings for postoperative nausea should be interpreted cautiously [7,8,26].

A subgroup analysis was conducted to investigate the effect of melatonin dose on the incidence of postoperative nausea (Figure 18). Preoperative melatonin did not significantly reduce the risk of postoperative nausea in either the 10 mg subgroup (RR 0.07; 95% CI 0.00 to 1.14; *p* = 0.062) or the 6 mg subgroup (RR 0.52; 95% CI 0.22 to 1.19; *p* = 0.057). Although the point estimates favored melatonin in both subgroups, the confidence intervals crossed the null value, and the findings should therefore be interpreted as exploratory (Figure 21).

##### Intraoperative Blood Loss

**Figure 22:**
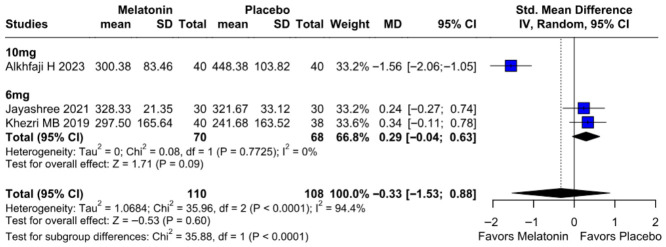
Subgroup analysis of intraoperative blood loss by melatonin dose. No significant differences were found in the 10 mg or 6 mg subgroups [23,25,26].

Subgroup analysis showed that preoperative melatonin did not significantly affect intraoperative blood loss in either the 10 mg (SMD −1.56) or 6 mg (SMD 0.29; *p* = 0.09) groups (Figure 22). The overall pooled effect was also non-significant (*p* = 0.60); however, the test for subgroup differences (*p* < 0.0001) showed a statistically significant subgroup difference between the subgroups. The apparent subgroup difference for intraoperative blood loss should be interpreted cautiously. Given the small number of studies, very high heterogeneity, and the influence of individual studies, this finding should not be considered evidence of a true dose-dependent effect.

#### 3.4.2. Risk of Bias

##### Pain Intensity

**Figure 23:**
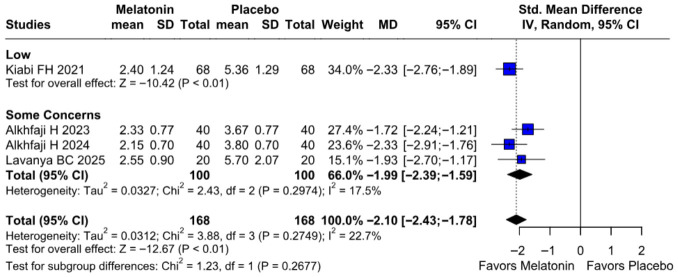
Forest plot of postoperative pain intensity: Melatonin vs. Placebo. Subgroup analysis by risk of bias demonstrates a significant overall reduction in pain with melatonin (pooled SMD = −2.10; 95% CI [−2.43, −1.78]; *p* < 0.01) with low heterogeneity (I^2^ = 22.7%) [8,23,24,27].

Melatonin significantly reduced postoperative pain intensity compared to placebo, with a pooled SMD of −2.10 (95% CI [−2.43, −1.78]; *p* < 0.01). Heterogeneity across the four studies was low and non-significant (I^2^ = 22.7%; *p* = 0.27). Subgroup analysis confirmed that the treatment effect remained consistent across studies with “Low” risk of bias (SMD = −2.33) and “Some Concerns” (SMD = −1.99), with no statistically significant difference between the two subgroups (*p* = 0.27) (Figure 23).

##### Postoperative Nausea

**Figure 24:**
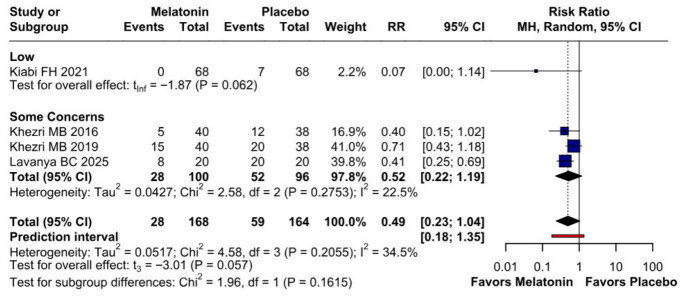
Forest plot of postoperative nausea: Melatonin vs. Placebo. The analysis shows a non-significant trend toward reduced nausea risk with melatonin (pooled RR = 0.49; 95% CI [0.23, 1.04]; *p* = 0.057). Subgroup analysis by risk of bias reveals no statistically significant differences between categories (*p* = 0.16) [7,8,26,27].

Melatonin showed a non-significant trend in reducing postoperative nausea compared to placebo (RR = 0.49; 95% CI [0.23, 1.04]; *p* = 0.057), with events occurring in 28/168 and 59/164 patients, respectively. Heterogeneity across the studies was moderate (I^2^ = 34.5%; *p* = 0.21). Subgroup analysis revealed no significant differences based on risk of bias (*p* = 0.16), while the wide prediction interval (0.18–1.35) suggests that the true effect in a future study could vary significantly and potentially cross the null line (Figure 24).

##### Intraoperative Blood Loss

**Figure 25:**
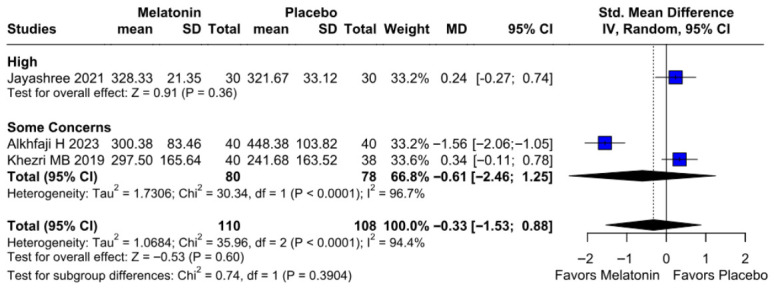
Forest plot of intraoperative blood loss. Melatonin vs. placebo showed no significant difference in blood loss (pooled SMD = −0.33; *p* = 0.60) [23,25,26].

Subgroup analysis for intraoperative blood loss showed no significant difference between melatonin and placebo (SMD = −0.33; 95% CI [−1.53, 0.88]; *p* = 0.60), with high heterogeneity observed (I^2^ = 94.4%). Results remained non-significant across both “High” risk (SMD = 0.24) and “Some Concerns” (SMD = −0.61) subgroups. The test for subgroup differences confirmed that study quality did not significantly influence the outcome (*p* = 0.39) (Figure 25).

### 3.5. Quality Assessment

**Figure 26:**
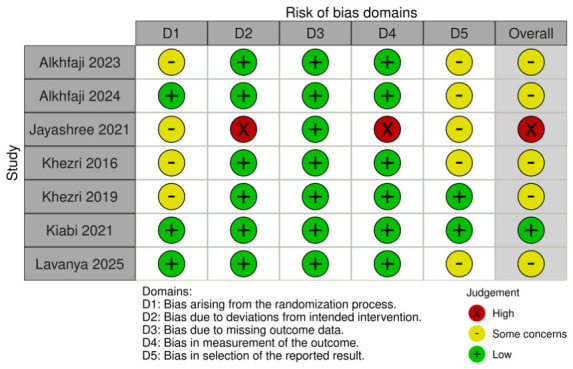
ROB assessment (RoB2.0) summary [7,8,23,24,25,26,27].

**Figure 27:**
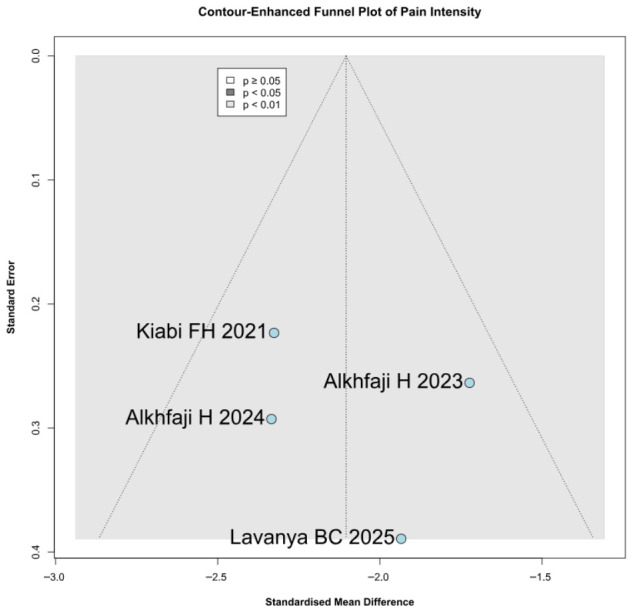
Contour-enhanced trim-and-fill funnel plot for postoperative pain levels. The plot illustrates individual study weights against point estimates [8,23,24,27].

**Figure 28:**
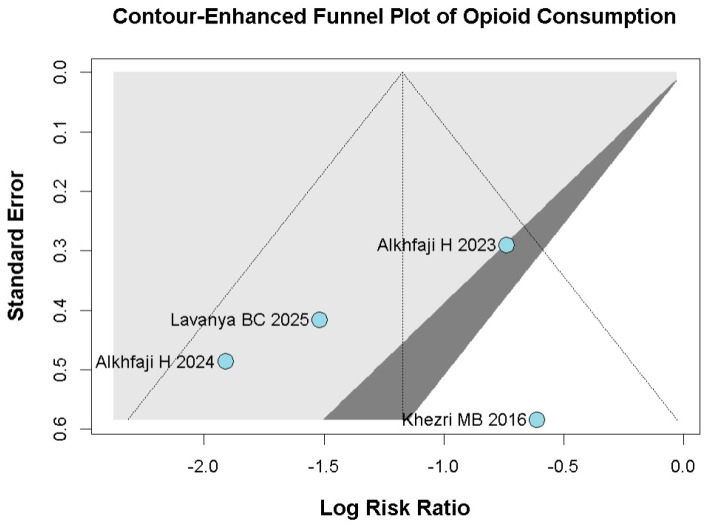
Contour-enhanced trim-and-fill funnel plot for opioid consumption. The plot illustrates individual study weights against point estimates [7,23,24,27].

**Figure 29:**
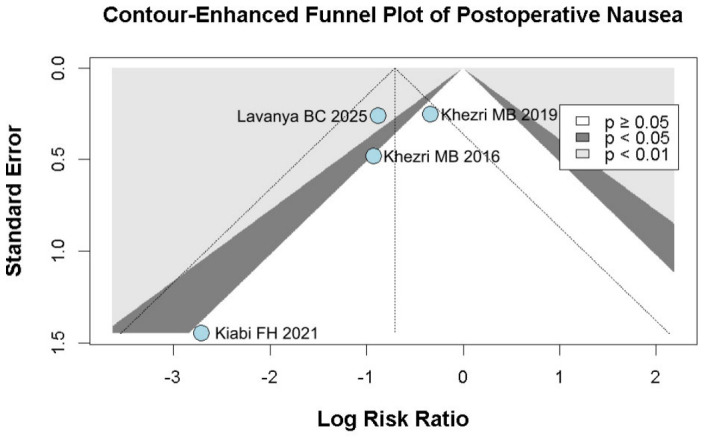
Contour-enhanced trim-and-fill funnel plot for postoperative nausea. The plot illustrates individual study weights against point estimates [7,8,26,27].

**Figure 30:**
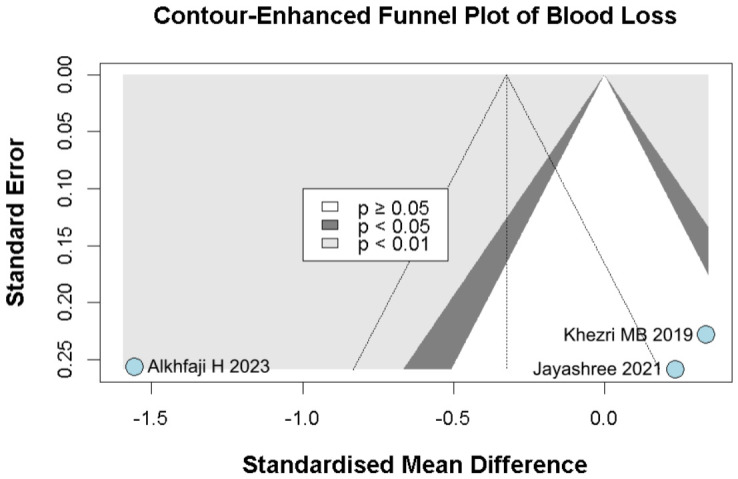
Contour-enhanced trim-and-fill funnel plot for intraoperative blood loss. The plot illustrates individual study weights against point estimates [23,25,26].

Among the seven included RCTs, five were assessed as having some concerns regarding risk of bias, one as having a low risk of bias, and one as having a high risk of bias based on the RoB2 tool. The detailed evaluation is presented in Figure 26. Funnel plots and trim-and-fill analyses were considered exploratory only because each outcome included fewer than 10 studies, and in some cases only 3–4 studies. Therefore, these analyses were not used to support firm conclusions regarding publication bias (Figure 27, Figure 28, Figure 29 and Figure 30).

## 4. Discussion

This updated systematic review and meta-analysis of seven RCTs demonstrates that preoperative melatonin is an effective adjunct in women undergoing cesarean section, with clinically meaningful reductions in postoperative pain intensity and opioid consumption [28,29]. These findings are supported by TSA under the selected assumptions; however, given the small number of trials and the possibility of overlapping study populations, TSA should be interpreted as supportive rather than definitive [12]. At the same time, melatonin showed a consistent but not conventionally statistically significant trend toward reducing postoperative nausea, while no effect was observed on intraoperative blood loss [30,31].

The most notable finding of this analysis is the magnitude and consistency of analgesic benefit associated with melatonin. The pooled standardized mean difference for postoperative pain (SMD −2.10) indicates a large effect size, suggesting that melatonin provides substantial improvement in postoperative comfort [28]. Importantly, this effect was accompanied by a significant reduction in opioid consumption (RR 0.31), highlighting a clinically relevant opioid-sparing effect [29,30]. In the context of cesarean section, where minimizing opioid exposure is particularly important due to maternal side effects and potential neonatal implications, this finding carries significant practical implications [32]. Reduced opioid exposure is clinically relevant after cesarean section because opioids may contribute to sedation, nausea, respiratory depression, delayed mobilization, and impaired maternal–infant interaction. The opioid-sparing effect observed in this meta-analysis is consistent with the broader clinical goal of reducing perioperative opioid exposure after cesarean section. Nevertheless, the included trials were small, and this finding should be confirmed in larger obstetric anesthesia trials using standardized analgesic protocols [29]. In the overlap-sensitivity analysis excluding Alkhfaji H 2023 [23], the opioid consumption estimate remained directionally favorable but no longer reached conventional statistical significance. This suggests that the opioid-sparing result may be sensitive to the inclusion of influential or potentially overlapping studies and should therefore be interpreted cautiously. Postoperative nausea and vomiting remain clinically important postoperative outcomes and are associated with patient discomfort, delayed oral intake, and reduced satisfaction. However, evidence that melatonin directly reduces PONV in cesarean patients remains limited and should be interpreted cautiously [31].

The dose-based subgroup analysis should be interpreted separately for pain, opioid consumption, and postoperative nausea. For postoperative pain, both the 6 mg and 10 mg melatonin subgroups showed statistically significant reductions compared with placebo. However, the test for subgroup differences was not statistically significant, indicating no evidence of a statistically significant difference between the two dose categories. Therefore, although both doses were associated with lower postoperative pain scores, the current evidence does not demonstrate a clear dose–response relationship between 6 mg and 10 mg melatonin.

For postoperative opioid consumption, the overall pooled analysis suggested a significant opioid-sparing effect of melatonin. However, when stratified by dose, neither the 6 mg nor the 10 mg subgroup reached statistical significance individually. This likely reflects the small number of studies and limited power within each subgroup. Therefore, the opioid-sparing effect should be interpreted primarily at the overall pooled level, and the current data do not provide reliable standalone evidence that either 6 mg or 10 mg independently reduces opioid consumption.

For postoperative nausea, neither the 6 mg nor the 10 mg subgroup reached statistical significance individually. Although the point estimates favored melatonin in both dose subgroups, the confidence intervals crossed the null value, and the results should therefore be considered exploratory. Consequently, the current evidence does not support firm dose-specific conclusions regarding the effect of melatonin on postoperative nausea.

For intraoperative blood loss, neither the 6 mg nor the 10 mg subgroup showed a statistically significant reduction compared with placebo, and the overall pooled effect was also non-significant. Although the test for subgroup differences was statistically significant, this finding should be interpreted with considerable caution because the analysis was based on a small number of studies, heterogeneity was very high, and individual studies may have had a disproportionate influence on the result. Therefore, the apparent difference between dose subgroups should not be interpreted as evidence of a true dose-dependent effect of melatonin on intraoperative blood loss.

The observed analgesic and opioid-sparing effects are biologically plausible and consistent with the known pharmacological profile of melatonin. Melatonin exerts antinociceptive effects through several complementary mechanisms. First, activation of MT1 and MT2 receptors in the central nervous system may modulate pain transmission at supraspinal and spinal levels. Second, melatonin appears to interact with endogenous opioid pathways, which may partly explain its opioid-sparing effect in the postoperative period. Third, melatonin reduces oxidative stress by directly scavenging reactive oxygen and nitrogen species and by enhancing antioxidant enzyme activity. Finally, it may attenuate surgical inflammation through downregulation of pro-inflammatory mediators such as interleukin-6, tumor necrosis factor-α, cyclooxygenase-related pathways, and nitric oxide signaling. These mechanisms are particularly relevant after cesarean section, where tissue injury, inflammatory activation, and central sensitization contribute to postoperative pain [33,34,35]. Experimental and clinical data suggest that melatonin modulates nociceptive processing through multiple mechanisms, including activation of MT1 and MT2 receptors, interaction with endogenous opioid pathways, and inhibition of pro-inflammatory cytokines [33,34]. Additionally, its antioxidant properties may attenuate surgical stress responses, further contributing to improved postoperative recovery [35]. The convergence of these mechanisms likely explains the consistent direction and magnitude of effect observed across the included trials.

The analysis of postoperative nausea revealed a trend toward benefit (RR 0.49), which narrowly missed conventional statistical significance (*p* = 0.057). TSA suggested a possible benefit for postoperative nausea under the selected assumptions. However, because the conventional meta-analysis narrowly missed statistical significance and the evidence base was small, this discordant TSA finding should be interpreted as exploratory rather than confirmatory in the conventional meta-analysis framework [12]. This discrepancy underscores the importance of complementing traditional meta-analytic methods with TSA, particularly in settings with limited sample sizes [12]. From a clinical perspective, even a modest reduction in postoperative nausea is relevant, as nausea and vomiting remain among the most distressing postoperative symptoms following cesarean delivery and can negatively impact maternal satisfaction and early recovery [31,36].

In contrast, no significant effect of melatonin was observed on intraoperative blood loss, and this outcome was characterized by substantial heterogeneity (I^2^ = 94%). Several factors may explain this finding. First, blood loss during cesarean section is influenced predominantly by surgical technique, uterotonic use, and obstetric factors rather than perioperative adjuncts such as melatonin [37,38]. Second, variability in measurement methods and reporting across studies likely contributed to the observed heterogeneity. Although the test for subgroup differences was statistically significant for intraoperative blood loss, this finding should be interpreted with great caution. The analysis was based on a small number of studies, heterogeneity was very high, and individual studies had substantial influence on the pooled estimate. Therefore, the observed subgroup difference should not be interpreted as definitive evidence of a dose-dependent effect.

An important strength of this study lies in its methodological rigor and critical appraisal of previously published evidence. Compared with earlier meta-analyses [11], the present study incorporated newly available RCTs while applying stricter inclusion criteria and enhanced data verification procedures. Notably, two studies included in the prior meta-analysis were excluded in our analysis—one being a conference abstract with insufficient extractable data and the other an untraceable study with an invalid DOI. The inclusion of such studies raises concerns regarding data reliability, reproducibility, and overall methodological transparency [14]. By excluding these potentially non-verifiable sources, our analysis minimizes the risk of bias and enhances the robustness and credibility of the pooled estimates. In addition, the application of advanced statistical techniques, including restricted maximum-likelihood random-effects modeling, LOO sensitivity analyses, Baujat diagnostics, and TSAs, further strengthens the validity of our findings [12,14,19]. The consistency of results across sensitivity analyses supports their stability, while the identification of influential studies did not materially alter the overall conclusions.

Although the present meta-analysis focused on maternal perioperative outcomes, obstetric and anesthetic interventions may also have implications for neonatal outcomes. However, none of the included RCTs reported long-term neonatal neurodevelopmental outcomes, including cerebral palsy. Therefore, no conclusion can be drawn regarding the effect of preoperative melatonin on cerebral palsy or long-term neonatal neurodevelopment. Future trials should include standardized neonatal safety outcomes and longer follow-up where feasible [39].

The number of participants required to support firm conclusions depends on the outcome, assumed clinically meaningful effect size, control event rate, heterogeneity, and desired type I and type II error thresholds. Therefore, no single sample-size threshold applies to all outcomes. In the present study, TSA was used to estimate whether the cumulative sample size approached or exceeded the required information size for selected outcomes. While TSA suggested that the available evidence may be sufficient for some efficacy outcomes under the adopted assumptions, the total sample size remains insufficient to support definitive conclusions regarding safety, neonatal outcomes, rare adverse events, and subgroup effects.

Nevertheless, several limitations should be acknowledged. First, the overall sample size remains relatively modest, with only seven RCTs and 552 participants included. Although TSA suggested that evidence may be sufficient for some outcomes, the limited number of trials reduces the precision of subgroup analyses and limits the ability to identify small but clinically relevant differences between melatonin doses, timing regimens, routes of administration, and anesthesia protocols. Therefore, findings related to secondary outcomes and subgroup effects should be interpreted cautiously. Second, the search did not include Embase, and the review was restricted to English-language full-text publications while excluding gray literature. These restrictions may increase the risk of language, publication, and availability bias, particularly given the small number of eligible RCTs. Third, potential overlap could not be fully excluded between some studies from the same author groups with highly similar baseline characteristics and intervention protocols. Fourth, postoperative pain is a time-dependent outcome, and differences in the timing of pain assessment across studies may have contributed to heterogeneity and may affect the interpretation of the large pooled SMD. Fifth, TSA results depend on assumptions regarding anticipated intervention effect and control event rate; therefore, TSA findings, particularly for postoperative nausea, should be interpreted as supportive and exploratory rather than definitive. Finally, funnel plots and trim-and-fill analyses were based on very few studies and should be considered exploratory only.

Additionally, the external validity of the findings may be limited by the geographic concentration of studies, with several trials conducted in similar healthcare settings. Differences in perioperative care pathways, analgesic protocols, and patient populations may influence the generalizability of the results. The exclusion of non-English studies and gray literature may also introduce a degree of publication bias, although formal assessment was limited by the small number of included studies [14].

From a clinical standpoint, the findings suggest that melatonin may be a promising adjunct to multimodal analgesia protocols for cesarean section. However, routine clinical integration cannot yet be recommended on the basis of the current evidence alone, given the modest sample size, risk-of-bias concerns, possible cohort overlap, variability in perioperative protocols, and limited neonatal safety data. Given its favorable safety profile, low cost, and ease of administration, melatonin represents an attractive adjunct for improving postoperative pain control while reducing reliance on opioids [28,40]. The favorable safety profile of melatonin has been reported in perioperative and non-perioperative settings. It is generally well tolerated, with mostly mild and transient adverse effects such as sleepiness, dizziness, headache, or nausea. Importantly, melatonin is not associated with respiratory depression, clinically relevant hemodynamic instability, or the dependence-related risks observed with opioids or sedative-hypnotics [32]. In the context of cesarean section, this is clinically relevant because reducing opioid exposure may improve maternal alertness, mobilization, breastfeeding, and early maternal–neonatal interaction. However, because pregnancy-specific and neonatal safety data remain comparatively limited, future trials should systematically report maternal adverse events, neonatal outcomes, breastfeeding outcomes, and sedation scores. However, routine implementation should be approached with consideration of existing institutional protocols and in conjunction with established analgesic strategies.

Future research should focus on large, high-quality, multicenter RCTs with standardized dosing regimens and clearly defined outcome measures. Particular attention should be given to neonatal outcomes, long-term maternal recovery, and optimal timing and dosing of melatonin administration. Further studies incorporating pharmacokinetic and pharmacodynamic analyses may also help elucidate the mechanisms underlying its clinical effects and optimize its use in obstetric anesthesia.

## 5. Conclusions

Preoperative melatonin may be a promising adjunct for reducing postoperative pain and opioid consumption in women undergoing cesarean section. However, given the modest evidence base and methodological limitations, further high-quality RCTs are needed before routine clinical implementation can be recommended.

## Figures and Tables

**Table 1:** Baseline characteristics of the included studies.

Characteristics/Study	Alkhfaji H 2023 [21]	Alkhfaji H 2024 [22]	Jayashree 2021 [26]	Khezri MB 2016 [7]	Khezri MB 2019 [23]	Kiabi FH 2021 [8]	Lavanya BC 2025 [27]
**Design**	RCT	RCT	RCT	RCT	RCT	RCT	RCT
**Country**	Multicenter	Multicenter	India	Iran	Iran	Iran	India
**No. Patients**	80	80	60	78	78	136	40
**Melatonin (M)**	40	40	30	40	40	68	20
**Placebo (P)**	40	40	30	38	38	68	20
Age *	M: 28.85 P: 30.20	M: 28.85 P: 30.20	M: 27.30 P: 27.80	M: 28.38 P: 28.63	M: 28.38 P: 28.63	N/A	M: 28.20 P: 28.40
Height *	M: 165.03 P: 162.30	M: 165.03 P: 162.30	N/A	M: 160.98 P: 161.6	M: 160.98 P: 161.6	N/A	N/A
Weight *	M: 77.60 P: 77.48	M: 77.60 P: 74.48	N/A	M: 72.45 P: 75.51	M: 72.45 P: 75.51	N/A	N/A
**Dosage**	10 mg	10 mg	6 mg	6 mg	6 mg	10 mg	10 mg

* Mean.

**Table 2:** Pain outcome extraction details used in the primary meta-analysis.

Study	Pain Scale	Pain Condition	Time Point
Alkhfaji 2023 [21]	VAS	Rest	closest 12–24 h value
Alkhfaji 2024 [22]	VAS	Rest	closest 12–24 h value
Khezri 2016 [7]	VPS	Rest	24 h
Kiabi 2021 [8]	VAS	Rest	24 h
Lavanya 2025 [27]	VAS	Rest	24 h

VAS, visual analogue scale; VPS, verbal pain scale. Only one pain time point per study was included in the primary pooled analysis to avoid double-counting participants.

## Data Availability

All the included data is available publicly in the databases used.
